# Production of Composite Zinc Oxide–Polylactic Acid Radiopaque Filaments for Fused Deposition Modeling: First Stage of a Feasibility Study

**DOI:** 10.3390/ma17122892

**Published:** 2024-06-13

**Authors:** Francesca Cherubini, Nicole Riberti, Anna Maria Schiavone, Fabrizio Davì, Michele Furlani, Alessandra Giuliani, Gianni Barucca, Maria Cristina Cassani, Daniele Rinaldi, Luigi Montalto

**Affiliations:** 1 Department of Construction, Civil Engineering and Architecture, Università Politecnica delle Marche, 60100 Ancona, Italy; f.cherubini@pm.univpm.it (F.C.); f.davi@staff.univpm.it (F.D.); 2 Department of Neurosciences, Imaging and Clinical Sciences, Università Gabriele D’Annunzio Chieti-Pescara, 66100 Chieti, Italy; nicole.riberti@unich.it; 3 Department of Science and Engineering of Materials, Environment and Urban Planning, Università Politecnica delle Marche, 60100 Ancona, Italy; a.m.schiavone@staff.univpm.it (A.M.S.); g.barucca@staff.univpm.it (G.B.); d.rinaldi@staff.univpm.it (D.R.); l.montalto@staff.univpm.it (L.M.); 4 Department of Odontostomatologic and Specialized Clinical Sciences, Università Politecnica delle Marche, 60100 Ancona, Italy; m.furlani@pm.univpm.it; 5 Department of Industrial Chemistry “Toso Montanari”, Alma Mater Studiorum Università di Bologna, 40126 Bologna, Italy; maria.cassani@unibo.it

**Keywords:** zinc oxide, radiopacity, polylactic acid, composite, nanocomposite, nanoparticles, additive manufacturing, extrusion

## Abstract

Three-dimensional printing technologies are becoming increasingly attractive for their versatility; the geometrical customizability and manageability of the final product properties are the key points. This work aims to assess the feasibility of producing radiopaque filaments for fused deposition modeling (FDM), a 3D printing technology, starting with zinc oxide (ZnO) and polylactic acid (PLA) as the raw materials. Indeed, ZnO and PLA are promising materials due to their non-toxic and biocompatible nature. Pellets of PLA and ZnO in the form of nanoparticles were mixed together using ethanol; this homogenous mixture was processed by a commercial extruder, optimizing the process parameters for obtaining mechanically stable samples. Scanning electron microscopy analyses were used to assess, in the extruded samples, the homogenous distribution of the ZnO in the PLA matrix. Moreover, X-ray microtomography revealed a certain homogenous radiopacity; this imaging technique also confirmed the correct distribution of the ZnO in the PLA matrix. Thus, our tests showed that mechanically stable radiopaque filaments, ready for FDM systems, were obtained by homogenously loading the PLA with a maximum ZnO content of 6.5% wt. (nominal). This study produced multiple outcomes. We demonstrated the feasibility of producing radiopaque filaments for additive manufacturing using safe materials. Moreover, each phase of the process is cost-effective and green-oriented; in fact, the homogenous mixture of PLA and ZnO requires only a small amount of ethanol, which evaporates in minutes without any temperature adjustment. Finally, both the extruding and the FDM technologies are the most accessible systems for the additive manufacturing commercial apparatuses.

## 1. Introduction

Further efforts are needed to develop, adapt, and rethink materials and production technologies in the direction of sustainability, eco compatibility, and waste reduction [1,2]. With reference to polymer-based products, additive manufacturing (AM) methods have clear advantages in terms of efficiency in material consumption and geometry customization at every level, from prototyping to industrial production, and in different fields from mechanics and electronics to medicine [3,4]. An appropriate choice of material can lead to a high level of recyclability at the end of the product’s life [5,6]. Numerous materials are available on the market today for different purposes from pure polymeric substances to composite structures for specific applications [7,8]. Among the various combinations, metal particles are added to an organic/polymer matrix to improve the mechanical properties and/or to achieve characteristics specific to polymers such as electrical and electronic characteristics, optical properties, and/or a desired radiation response. A class of materials intended for radioprotection and shielding dedicated to treating different wavelengths and types of radiation, from deep ultraviolet to X-rays and gamma rays, is emerging [9,10]. Recent progress has been made using a barium-based filler in an organic matrix in different forms such as barium sulfate or barium oxide mixed with PLA/ethylene vinyl acetate (EVA) copolymer [11,12], showing a good formability together with strong mechanical properties and good radio absorption. Attempts are underway with tungsten dispersed in PLA to achieve gamma radiation attenuation performance [13]. Lead is also used together with PEG and PLA to produce a radiation shielding material [10,14]. Promising results have come from these combinations of organic and metallic materials [15,16]. 

However, the eco-compatibility of compounds is not always ensured for both the metallic and polymeric parts. Care must be taken in the design of the entire manufacturing process from compound synthesis to mechanical processing to final geometry, along with an accurate life cycle assessment, to reduce the environmental impact and potential toxicity of composite materials [17,18]. 

In this work, a safer solution is proposed; it includes materials and processes which merge accessibility, reduced impact, and cost. As fused deposition modeling is one of the most accessible and sustainable production processes, the feasibility of using composite filaments for 3D printers was first approached by Chong et al. [19], taking into account that non-toxic zinc oxide (ZnO) has a great ability to be synthetized via eco-friendly processes [20]. 

Polylactic acid (PLA) is a polyester with several promising properties: it has good mechanical [12] and chemical properties; it is biodegradable, biocompatible, and thermoplastic [21,22,23]. Thanks to these characteristics, it is used for various applications in the biomedical and technological fields [21,22,23]. As they are biopolymers produced from natural resources, at the end of their lives, PLA products mostly end up in landfills or compost [23]. Previous studies have analyzed the existing life cycle assessments (LCAs) for PLA and have highlighted that it is possible to make materials with reduced environmental impact by optimizing the PLA conversion process, which is the most energy-intensive stage in the life cycle of PLA [23]. This aspect makes PLA a particularly advantageous material from an environmental point of view. At present, PLA is the most readily available filament used for 3D printing with the fused deposition modelling (FDM) method, being the simplest and the most common method related to additive manufacturing technologies [22,24]. 

The nanoparticles in composites can be homogeneously distributed in a polymer matrix, which provides greater interfacial interactions [25]. The addition of specific nanofillers into the PLA matrix can lead to major improvements in PLA characteristics (mechanical, thermal, barrier, etc.) [26]. Among the various nanoparticles, zinc oxide (ZnO) is particularly interesting because of its properties and production processes. It is an inorganic compound and an important semiconductor material. From an environmental point of view, it is also low in toxicity, biocompatible, and biodegradable. It is largely accessible in different crystalline phases and structural forms; it shows good mechanical and thermal properties such as hardness and a high plastic penetration level. Due to its density and atomic number, zinc oxide has great absorption of short-wavelength radiation, while in monocrystal form, it has good transparency in the optical range; in fact, this semiconductor material has the potential to be used as a radiation-shielding material [27,28], and, when properly doped, zinc oxide is used as scintillator too. In addition to these functional and physical properties, ZnO has interesting characteristics in terms of biocompatibility and toxicity [28,29]. Largely used in cosmetics for skin recovery and protection, ZnO finds a place in a number of applications related to the biomedical field, from tissue integration to drug delivery and biosensors [30]. Together with its biocompatibility, the antibacterial and antimicrobial activities of ZnO have been studied and exploited for medical purposes [31,32,33], placing this material amongst the safest ones. 

The incorporation of ZnO into a PLA matrix was found to potentially accelerate the degradation of PLA but also reinforce the mechanical properties [34,35,36,37]. From the point of view of optical properties, most polymers are considered transparent to X-rays, while zinc oxide (ZnO) has the ability to be radiopaque [38]. 

This property is interesting because ZnO-reinforced polymer composites could be used to fabricate radiopaque materials as an alternative to Pb and Ba for absorbing or blocking radiation. In particular, Noor Azman et al. [28] investigated the X-ray-attenuation capability of different types of nanofiber mats at low X-ray energies with scanning electron microscopy (SEM), finding that the nanofiber mat with the highest percentage of n-ZnO among those analyzed had the best X-ray attenuation capabilities.

For these reasons, PLA–ZnO nanocomposites are gaining attention for many applications [26,38]; in particular, zinc oxide can be “green synthesized” and showed a good biodegradability in tissue and in combination with PLA [37]. In order to design the extrusion of PLA-ZnO composite films, Murariu et al. [39] studied the dissolution of PLA with a certain percentage of rod-like ZnO nanoparticles previously surface treated with a specific silane, revealing that this particular addition of treated filler leads to a very significant enhancement in the PLA–ZnO properties. In addition, the study of Murariu et al. [26] clarified the positive effects of mixing preliminarily ZnO in PLA and its use as a solid additive in an alternative manufacturing technique. Recently, attention was also dedicated to improving the production of PLA-ZnO composites in the additive manufacturing of PLA-ZnO composite material, with particular attention on the FDM method [37].

The present study, for the first time to the best of our knowledge, demonstrates the feasibility of obtaining a printable filament—by appropriately mixing ZnO nanoparticles to the PLA matrix—with the aim of obtaining manageable optical properties aimed at the absorption of X-ray radiation. This was achieved through a tailor-made process where each step was carefully chosen and adapted to achieve the goal of keeping the entire procedure—from material choice to manufacturing process—completely safe within a cost-effective environment, also allowing the possibility of recycling the final product.

## 2. Materials and Methods

The PLA and the ZnO particles used in this work were both commercial. The PLA pellets had the following characteristics, as declared by the producer (FELFIL, Torino, Italy): extrusion temperature: 160–175 °C; printing temperature: 185–195 °C [40]. The commercial ZnO was provided by ELEMENTAL SRL (Oradea, Romania) and was used as a nanofiller. 

Ethanol and a FELFIL EVO commercial desk extruder (FELFIL, Torino, Italy) were used for preparing and processing the mixture, while for the characterization of the materials, Bruker AXS D8 Advance X-ray Diffractometer (XRD, Karlsruhe, Germany), Zeiss Supra 40 scanning electron microscopy (SEM, Oberkochen, Germany), and Bruker Skyscan 1174 microtomography (Micro-CT, Kontich, Belgium) systems were used.

The experimental process was carried out in four steps: 1. characterization of the incoming ZnO powder (Supplementary Materials); 2. preparation of the mixture; 3. extrusion of the mixture for producing the printable filaments; 4. characterization of the PLA-ZnO filaments, obtaining information on ZnO distribution and radiopacity characteristics.

### 2.1. Preparation of Mixture

Four samples were prepared. For each sample, 50 g of PLA pellets and 4 g of ZnO were mixed. Both PLA and ZnO were weighed with a scientific balance with an accuracy of ±0.0001 g; the measured data are shown in Table 1. The PLA-ZnO mixture was mixed in beakers using ethanol at room temperature to slightly moisten the surface of the PLA, allowing the ZnO particles to be absorbed by the polymer surface. The use of ethanol is one of the key points in this study, because it is a compound that is not only economical but also has a low environmental impact. With this method, the PLA was loaded with a ZnO content equal to a maximum of 6.5% by weight (nominal); this upper limit depended on the surface area of the pellets, i.e. when the entire surface of the PLA pellet is covered by the ZnO powder, the upper limit is reached. Before processing, the mixture was dried at room temperature to completely evaporate the ethanol. The dried mixture was weighed to evaluate the amount of ZnO adsorbed and the percentage obtained. The weights and percentages of PLA and ZnO are reported for the different phases in Table 1.

### 2.2. Processing the Mixture to the Filament Shape

**Figure 1:**
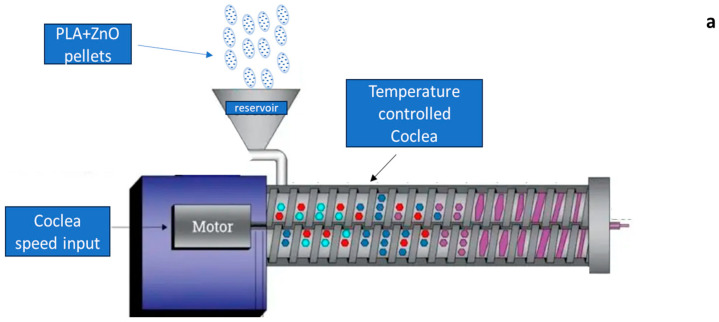

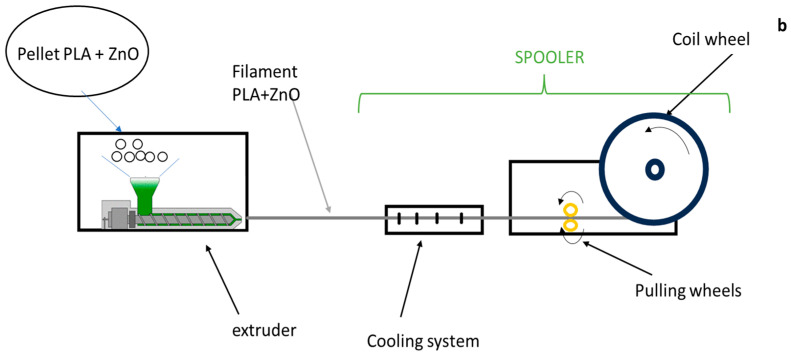
(**a**) Extruder scheme; (**b**) filament extrusion layout.

A commercial FELFIL EVO desk extruder (FELFIL, Torino, Italy) was used to produce the filaments. In addition to the ZnO/PLA percentage optimization in the incoming mixture (Table 1), the extrusion parameters were optimized in order to obtain a stable filament. The prepared mixture was entirely placed into the tank of the extrusion device to allow the system to feed the cochlea coherently with its geometry and rotation speed. Four samples, with an average diameter of 1.1 mm, were produced with stabilized geometric and mechanical features, ready for successive characterization. The scheme and the layout of the filament production route are shown in Figure 1.

### 2.3. SEM Observations and EDS Analysis

The incoming ZnO nanoparticles were analyzed by SEM equipped with energy-dispersion spectroscopy (EDS) for morphology and dimension assessment and chemical analysis. A Zeiss Supra 40 (ZEISS, Oberkochen, Germany) was used for the purpose; for the morphology evaluation, the scanning electron microscope had a magnification from 5.00 KX to 100.00 KX, with the electron gun set to 5 kV; secondary electrons were acquired. The EDS was carried out at20 kV in the EDS Microanalysis Bruker Quantax 200-Z10 (Bruker Nano GmbH, Berlin, Germany) of the Zeiss microscope. 

The extruded composite filaments were then analyzed via SEM TESCAN Vega3 (TESCAN, Brno, Czech Republic) for evaluating the proper dispersion of the ZnO particles over the PLA matrix; in this case, the following working parameters were set: WD = 15 ÷ 20 mm, HV = 20 kV, magnification of 1.00 KX, and high scanning speed to avoid excessive degradation of the PLA during image acquisition.

### 2.4. XRD for ZnO Characterization

ZnO structural assessment was performed using a BRUKER AXS D8 Advance X-ray Diffractometer (XRD) (BRUKER, Karlsruhe, Germany) for crystallinity and phase recognition. The Bragg–Brentano configuration was used, with an angular range of 2θ between 30 and 80 degrees; the resolution was set to 0.02 degrees per step, with a time duration of 2 s per step.

### 2.5. Micro-CT Characterization of the Final Filaments

The three-dimensional (3D) analysis of the final filaments was performed via X-ray Micro-CT. This method allowed for assessing the overall distribution of the ZnO particles inside the PLA matrix and the X-ray absorption properties of the final product. 

Two commercial lab-based Micro-CT systems were used in this study. 

The first one was a Bruker-Skyscan 1174 (BRUKER, Antwerp, Belgium), which was set with a voltage of 50 kV, and a beam current of 800 μA. For each test, the same parameters were used: pixel size = 6.5 μm; 0.25 mm Al filter; exposure time = 2 s per projection. Samples were scanned over 180°, using a 0.3° scan step. After the tomographic scan, the reconstruction phase was executed with the Bruker 3D SUITE software package, setting Smoothing algorithm: 3.0; Ring Artifact Reduction: 3.0; and Beam Hardening correction: 15%. 

The second lab-based Micro-CT system was a Metro-Tom 1500 ZEISS (Carl Zeiss, Germany) using a 120 kV and 104 μA X-ray source with an Al filter of 0.5 mm. In this case, a pixel size of 11.7 μm was used, imaging all samples together in the same field of view. The image reconstruction was automatically performed by Metro-Tom software. Afterwards, all images were elaborated with Dragonfly software (Vers. 2022.2; Object Research Systems, Montréal, QC, Canada) [41].

## 3. Results

The present study established a procedure that simulates a possible production route, including the inspection and quality control of the incoming raw materials. Therefore, an accurate analysis of the starting materials was mandatory and was achieved using SEM, EDS and XRD analyses. The characterization of the incoming raw materials is provided in the Supplementary Materials.

### 3.1. SEM Observation of PLA+ZnO Extruded Filaments

**Figure 2:**
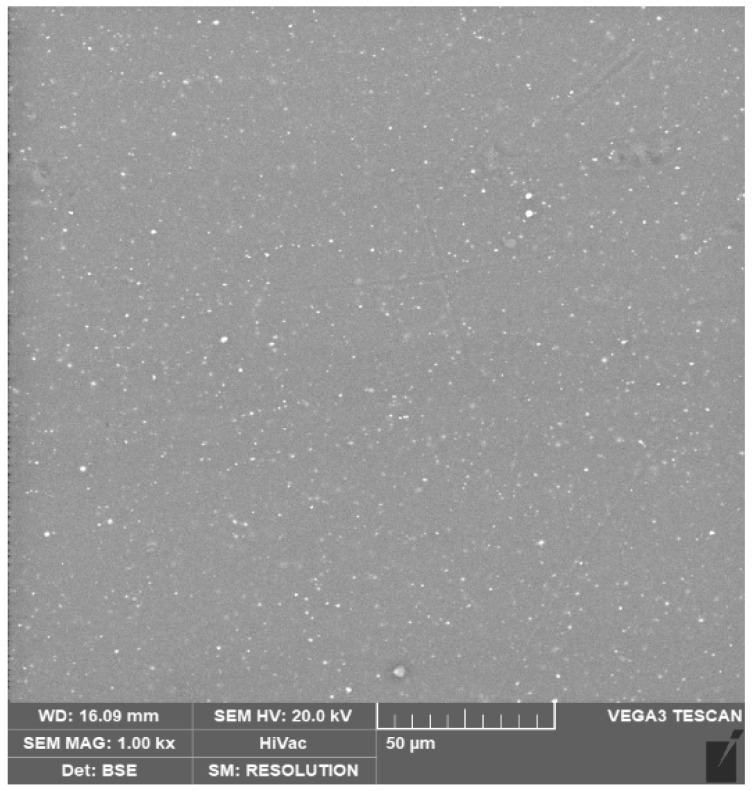
SEM image of the filament surface. The bright spots over the grey background represent the ZnO particles that were homogeneously distributed over the PLA matrix (the grey background). Some agglomerates, not larger than the ones present in the raw powder, were also detected.

The distribution of zinc oxide (white particles) inside a filament (grey base) is shown in Figure 2. The figure shows the homogeneous ZnO particle distribution throughout the sample. 

### 3.2. Bruker Skyscan 1174 Micro-CT Analysis of the Filament

**Figure 3:**
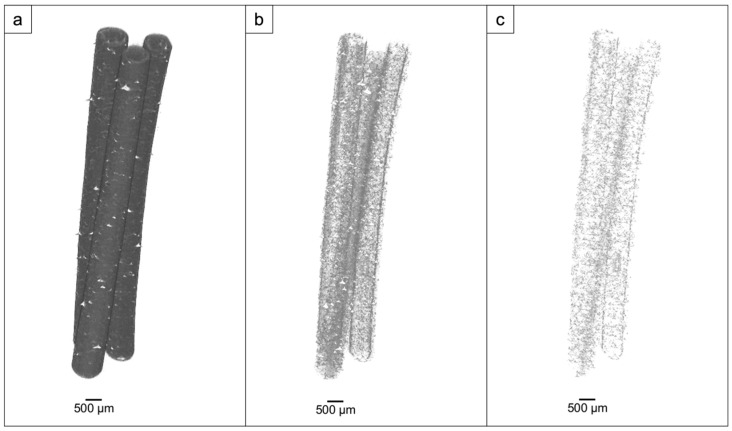
Test 4: 3D reconstructions, with opacity set at different window levels: 30–255 (**a**), 100–255 (**b**), 130–255 (**c**).

For the micro-CT tests using the Bruker Skyscan 1174, three portions of each sample, cut from the initial, the central, and the final portions of the extruded filaments, were acquired. The length of each portion was 15 mm. The aim of this test was to assess in 3D the density and the distribution of the ZnO in each PLA filament. As a representative example, the results obtained in each portion of Test 4 are reported in Figure 3.

In the reconstructed images, the grey tones were linked to the absorption properties of the analyzed samples. Increasing the segmentation threshold meant making the grey value vanish below a certain level; in this way, it was possible to hide the PLA and display only ZnO particles and aggregates. Therefore, only the ZnO particles are shown in Figure 3c. They exhibit a greater density, as shown by the white tones, thus showing that they possessed enhanced absorbency. These entities (ZnO nanoparticles and aggregates) were found to be dispersed throughout the entirety of the thread in all three segments. 

To evaluate whether the radiopacity was homogeneous in all samples, their absorption properties were measured via grey-level histograms. From each of them, six histograms were extracted: two for each of the three portions of the sample (upper, central, and lower).

**Figure 4:**
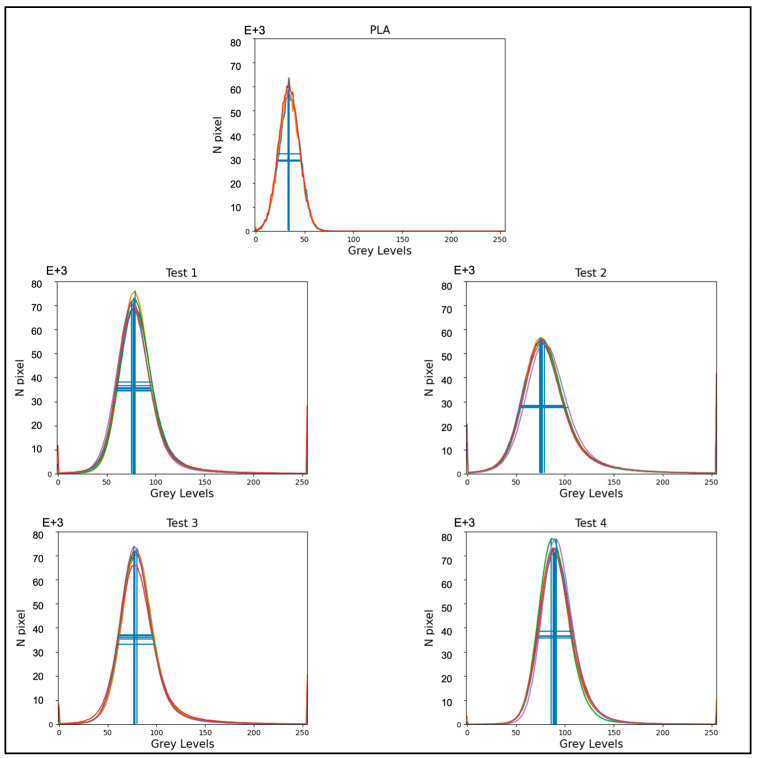
The grey-level histograms representing the overall density of the control (PLA) filament (central top graph) and test filaments. The lines marked inside the peaks represent the amplitude (vertical lines) and the full width at half maximum (FWHM—horizontal lines). Each color line represents the histogram from a single portion of each Test sample.

Figure 4 shows that the peaks of the histograms for the selected portions approximately overlap for each sample: this indicates that the distribution of grey levels was uniform along all filaments. Therefore, it was found that ZnO was homogeneously distributed in the PLA matrix. The images were reconstructed in 8 bits (0–255 grey levels). The density of the material is represented by these levels, mainly concentrated in the range 0–150, in Figure 4. The average grey level for each peak indicates the most relevant shade of grey for each portion of the filament. As we moved towards higher values, where the pixels were brighter, we saw how the introduction of ZnO into the filament increased its radiopacity and therefore the density of the filament itself (average grey level: 76–88) compared to pure PLA filament (Figure 4—top graph: PLA), which showed an average grey level of 34.

**Figure 5:**
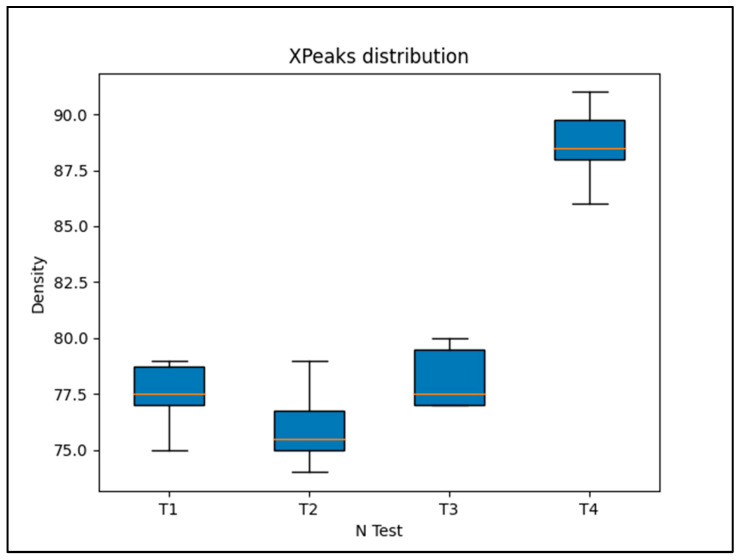
Box plots depict the extracted mean grey level through each quartile as a function of the selected groups of samples: Test 1, Test 2, Test 3, and Test 4. The horizontal red lines represent the median density values in terms of grey level (full scale: 0–255).

Box plots (Figure 5) graphically depict the extracted mean grey level through each quartile as a function of the selected group of samples: Test 1, Test 2, Test 3, and Test 4. The physical density, expressed as a grey level, agrees with the percentages of ZnO entering the extrusion process (ZnO in incoming mixture (%)), as shown in the last column in Table 1.

### 3.3. Metro-Tom ZEISS Micro-CT for Filament Evaluation

Metro-Tom micro-CT was used for acquiring the complete sample population in a single shot. Mismatches in sample radiopacities were clearly visible in the same 3D acquisition, avoiding the risk of noise from possible uncontrolled variations in the acquisition parameters. 

Dragonfly software was used for image processing and data analysis (Vers. 2022.2; Object Research Systems, Montreal, QC, Canada). 

**Figure 6:**
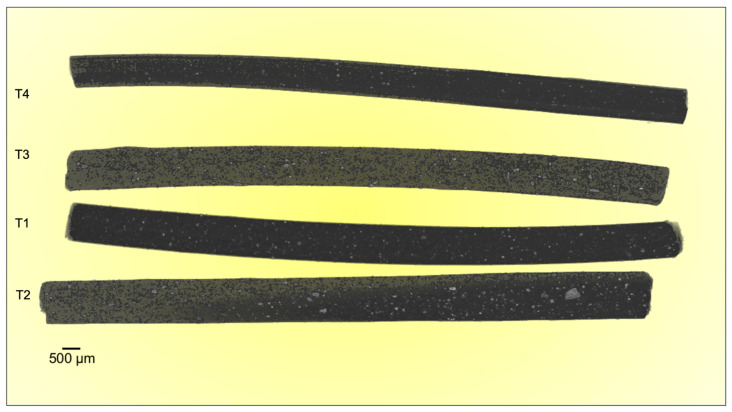
Metro-Tom computed microtomographic acquisition of the entire test filaments.

The 3D images observed in Figure 6 were rendered at 16 bits (corresponding to 65,536 grey levels). The ZnO aggregates can be clearly observed in the form of white spots distributed along the entire body of each filament. Furthermore, Figure 6 shows no inhomogeneity in X-ray radiopacity or thus in the distribution of ZnO nanoparticles along the filaments, indicating that the production of the composite occurred in a fully stabilized manner, in accordance with the Bruker Skyscan Micro-CT results (Figure 3).

## 4. Discussion

The experimental results demonstrate the reliability of this new production route to obtain filaments, characterized by homogenous radiopacity and based on safe, economic, and accessible raw materials and technologies. This was achieved by mixing ZnO nanoparticles and PLA pellets using ethanol, followed by the extrusion of composite materials with homogeneous characteristics. Indeed, the SEM analysis of the extruded samples showed that the zinc oxide nanoparticles were uniformly distributed over the PLA matrix without creating agglomerates larger than the ones present in the raw powder (Figure 2). These data were confirmed by micro-CT measurements; indeed, the grey level measured along each filament (Figure 4) revealed that the x position (i.e. radiopacity) and the full width at half maximum between the different zones of the same test were similar, indicating a uniform distribution of the ZnO nanoparticles, which raised the radiopacity uniformly across each filament. However, as shown in Figure 3, the x value of the peak was not directly determined by the ZnO clusters but by the homogeneous distribution of ZnO nanoparticles along each filament. In fact, since the ZnO was in the form of nanoparticles, the radiopacity induced by the introduction of ZnO into the composite material was more an expression of the homogeneous distribution of the nanoparticles rather than of the rare presence of ZnO clusters. This was further confirmed by the reduced dimension of the box plots, showing low density variability between the 25th and the 75th percentile (Figure 5) and on a larger scale, by the uniform grey level exhibited in 3D in each filament (Figure 6). 

These data demonstrate that the procedure led to a proper ZnO distribution. Furthermore, microtomography provided evidence that the composite filaments acquired radioabsorption properties and that the radiopacity was coherent with the ZnO content. Thus, it was demonstrated that the proposed processing route achieves the purpose of producing radiopaque filaments suitable for FDM solutions. 

This study opens the way for designing and building customized products with desired optical/radiation properties and specific geometries for various applications at the laboratory sample level, avoiding the complexity and costs of large industrial series.

This research is the first step in a broader feasibility study for the production of innovative filaments for FDM printing technologies. This research is ongoing, and the optimization of the procedure for producing radiopaque objects from these composite filaments will be the subject of follow-up studies. In fact, the next step in the overall study will be to test the smoothness of printing these filaments to meet FDM requirements.

In conclusion, the production of filaments with customized properties in terms of radioabsorption for fusion deposition modeling was achieved in a reliable and affordable way, completely in line with an eco-friendly environment, from raw components to processes and technologies.

## Figures and Tables

**Table 1:** PLA and ZnO weights and percentages at different steps before extrusion processing.

Sample	PLA (g)	ZnO (g)	Dried PLA + ZnO (g)	ZnO in Incoming Mixture (g)	ZnO in Incoming Mixture (%)
Test 1	50.1619	4.0537	52.6027	2.4408	4.6401%
Test 2	50.1297	4.0452	52.2916	2.1619	4.1343%
Test 3	50.0379	4.0060	53.1898	3.1519	5.9258%
Test 4	50.0050	4.0307	53.4799	3.4749	6.4976%

## Data Availability

The data presented in this study are available on request from the corresponding author due to large data size.

## Supplementary Materials

The following supporting information can be downloaded at: https://www.mdpi.com/article/10.3390/ma17122892/s1, Figure S1: SEM images of ZnO powder; Figure S2: EDS microanalysis of the ZnO powder; Figure S3: XRD spectrum of the ZnO powder; Table S1: EDS microanalysis data of ZnO powder.

## References

[1] Kirchherr J., Yang N.-H.N., Schulze-Spüntrup F., Heerink M.J., Hartley K. (2023). Conceptualizing the Circular Economy (Revisited): An Analysis of 221 Definitions. Resour. Conserv. Recycl..

[2] D’Amato D., Korhonen J. (2021). Integrating the Green Economy, Circular Economy and Bioeconomy in a Strategic Sustainability Framework. Ecol. Econ..

[3] Saleh Alghamdi S., John S., Roy Choudhury N., Dutta N.K. (2021). Additive Manufacturing of Polymer Materials: Progress, Promise and Challenges. Polymers.

[4] Ligon S.C., Liska R., Stampfl J., Gurr M., Mülhaupt R. (2017). Polymers for 3D Printing and Customized Additive Manufacturing. Chem. Rev..

[5] Thormark C. (2006). The Effect of Material Choice on the Total Energy Need and Recycling Potential of a Building. Build. Environ..

[6] Cordella M., Alfieri F., Sanfelix J., Donatello S., Kaps R., Wolf O. (2020). Improving Material Efficiency in the Life Cycle of Products: A Review of EU Ecolabel Criteria. Int. J. Life Cycle Assess..

[7] Rajak D.K., Pagar D.D., Kumar R., Pruncu C.I. (2019). Recent Progress of Reinforcement Materials: A Comprehensive Overview of Composite Materials. J. Mater. Res. Technol..

[8] Rajak D., Pagar D., Menezes P., Linul E. (2019). Fiber-Reinforced Polymer Composites: Manufacturing, Properties, and Applications. Polymers.

[9] Kickelbick G. (2003). Concepts for the Incorporation of Inorganic Building Blocks into Organic Polymers on a Nanoscale. Prog. Polym. Sci..

[10] More C.V., Alsayed Z., Badawi M.S., Thabet A.A., Pawar P.P. (2021). Polymeric Composite Materials for Radiation Shielding: A Review. Environ. Chem. Lett..

[11] Suman S.K., Mondal R.K., Kumar J., Dubey K.A., Kadam R.M., Melo J.S., Bhardwaj Y.K., Varshney L. (2017). Development of Highly Radiopaque Flexible Polymer Composites for X-Ray Imaging Applications and Copolymer Architecture-Morphology-Property Correlations. Eur. Polym. J..

[12] Yang J., Wang C., Shao K., Ding G., Tao Y., Zhu J. (2015). Morphologies, Mechanical Properties and Thermal Stability of Poly(Lactic Acid) Toughened by Precipitated Barium Sulfate. Russ. J. Phys. Chem. A.

[13] Abulyazied D.E., Issa S.A.M., Alrowaily A.W., Saudi H.A., Zakaly H.M.H., Ali E.S. (2023). Polylactic Acid Tungsten Trioxide Reinforced Composites: A Study of Their Thermal, Optical, and Gamma Radiation Attenuation Performance. Radiat. Phys. Chem..

[14] Yilmaz M., Pekdemir M.E., Özen Öner E. (2023). Evaluation of Pb Doped Poly(Lactic Acid) (PLA)/Poly(Ethylene Glycol) (PEG) Blend Composites Regarding Physicochemical and Radiation Shielding Properties. Radiat. Phys. Chem..

[15] Gomez-Romero P. (2001). Hybrid Organic-Inorganic Materials—In Search of Synergic Activity. Adv. Mater..

[16] Carné A., Carbonell C., Imaz I., Maspoch D. (2011). Nanoscale Metal–Organic Materials. Chem. Soc. Rev..

[17] Huang S.H., Liu P., Mokasdar A., Hou L. (2013). Additive Manufacturing and Its Societal Impact: A Literature Review. Int. J. Adv. Manuf. Technol..

[18] Tancini F., Wu Y., Schweizer W.B., Gisselbrecht J., Boudon C., Jarowski P.D., Beels M.T., Biaggio I., Diederich F. (2012). 1,1-Dicyano-4-[4-(Diethylamino)Phenyl]Buta-1,3-dienes: Structure–Property Relationships. Eur. J. Org. Chem..

[19] Chong W.J., Pejak Simunec D., Trinchi A., Kyratzis I., Li Y., Wright P., Shen S., Sola A., Wen C. (2024). Advancing the Additive Manufacturing of PLA-ZnO Nanocomposites by Fused Filament Fabrication. Virtual Phys. Prototyp..

[20] Agarwal H., Venkat Kumar S., Rajeshkumar S. (2017). A Review on Green Synthesis of Zinc Oxide Nanoparticles—An Eco-Friendly Approach. Resour.-Effic. Technol..

[21] Murariu M., Dubois P. (2016). PLA Composites: From Production to Properties. Adv. Drug Deliv. Rev..

[22] Amirov A., Omelyanchik A., Murzin D., Kolesnikova V., Vorontsov S., Musov I., Musov K., Khashirova S., Rodionova V. (2022). 3D Printing of PLA/Magnetic Ferrite Composites: Effect of Filler Particles on Magnetic Properties of Filament. Processes.

[23] Rezvani Ghomi E.R., Khosravi F., Saedi Ardahaei A.S., Dai Y., Neisiany R.E., Foroughi F., Wu M., Das O., Ramakrishna S. (2021). The Life Cycle Assessment for Polylactic Acid (PLA) to Make It a Low-Carbon Material. Polymers.

[24] Bettini P., Alitta G., Sala G., Di Landro L. (2017). Fused Deposition Technique for Continuous Fiber Reinforced Thermoplastic. J. Mater. Eng. Perform..

[25] Mallick P.K. (2007). Fiber-Reinforced Composites.

[26] Murariu M., Benali S., Paint Y., Dechief A.-L., Murariu O., Raquez J.-M., Dubois P. (2021). Adding Value in Production of Multifunctional Polylactide (PLA)–ZnO Nanocomposite Films through Alternative Manufacturing Methods. Molecules.

[27] Bikiaris N.D., Koumentakou I., Samiotaki C., Meimaroglou D., Varytimidou D., Karatza A., Kalantzis Z., Roussou M., Bikiaris R.D., Papageorgiou G.Z. (2023). Recent Advances in the Investigation of Poly(Lactic Acid) (PLA) Nanocomposites: Incorporation of Various Nanofillers and Their Properties and Applications. Polymers.

[28] Noor Azman N.Z., Wan Mohamed W.F.I., Ramli R.M. (2022). Synthesis and Characterization of Electrospun N-ZnO/n-Bi_2_O_3_/Epoxy-PVA Nanofiber Mat for Low X-Ray Energy Shielding Application. Radiat. Phys. Chem..

[29] Nain V., Kaur M., Sandhu K.S., Thory R., Sinhmar A. (2020). Development, Characterization, and Biocompatibility of Zinc Oxide Coupled Starch Nanocomposites from Different Botanical Sources. Int. J. Biol. Macromol..

[30] Zhang Y., Nayak T., Hong H., Cai W. (2013). Biomedical Applications of Zinc Oxide Nanomaterials. Curr. Mol. Med..

[31] Wiesmann N., Mendler S., Buhr C.R., Ritz U., Kämmerer P.W., Brieger J. (2021). Zinc Oxide Nanoparticles Exhibit Favorable Properties to Promote Tissue Integration of Biomaterials. Biomedicines.

[32] Oleshko O., Husak Y., Korniienko V., Pshenychnyi R., Varava Y., Kalinkevich O., Pisarek M., Grundsteins K., Pogorielova O., Mishchenko O. (2020). Biocompatibility and Antibacterial Properties of ZnO-Incorporated Anodic Oxide Coatings on TiZrNb Alloy. Nanomaterials.

[33] Mahalakshmi S., Hema N., Vijaya P.P. (2020). In Vitro Biocompatibility and Antimicrobial Activities of Zinc Oxide Nanoparticles (ZnO NPs) Prepared by Chemical and Green Synthetic Route—A Comparative Study. Bionanoscience.

[34] Qu M., Tu H., Amarante M., Song Y., Zhu S.S. (2014). Zinc Oxide Nanoparticles Catalyze Rapid Hydrolysis of Poly(Lactic Acid) at Low Temperatures. J. Appl. Polym. Sci..

[35] Hussain M., Khan S.M., Shafiq M., Abbas N. (2023). Mechanical and Degradation Studies on the Biodegradable Composites of a Polylactic Acid Matrix Reinforced by Tricalcium Phosphate and ZnO Nanoparticles for Biomedical Applications. JOM.

[36] Ghozali M., Fahmiati S., Triwulandari E., Restu W.K., Farhan D., Wulansari M., Fatriasari W. (2020). PLA/Metal Oxide Biocomposites for Antimicrobial Packaging Application. Polym. -Plast. Technol. Mater..

[37] Chong W.J., Shen S., Li Y., Trinchi A., Pejak D., (Louis) Kyratzis I., Sola A., Wen C. (2022). Additive Manufacturing of Antibacterial PLA-ZnO Nanocomposites: Benefits, Limitations and Open Challenges. J. Mater. Sci. Technol..

[38] Pantani R., Turng L. (2015). Manufacturing of Advanced Biodegradable Polymeric Components. J. Appl. Polym. Sci..

[39] Murariu M., Paint Y., Murariu O., Raquez J., Bonnaud L., Dubois P. (2015). Current Progress in the Production of PLA–ZnO Nanocomposites: Beneficial Effects of Chain Extender Addition on Key Properties. J. Appl. Polym. Sci..

[40] Felfil. https://felfil.com/full-extrusion-system/?v=5ea34fa833a1.

[41] Makovetsky R., Piche N., Marsh M. (2018). Dragonfly as a Platform for Easy Image-Based Deep Learning Applications. Microsc. Microanal..

