# Supplementary material for: Production of Composite Zinc Oxide–Polylactic Acid Radiopaque Filaments for Fused Deposition Modeling: First Stage of a Feasibility Study
Source: Materials (Basel). 2024 Jun 13;17(12):2892. doi: 10.3390/ma17122892 (PMC11204736; doi:10.3390/ma17122892)
Supplement: Supplementary file 1 [file materials-17-02892-s001.zip › materials-2998140-supplementary.pdf]

Supplementary Materials

Scanning Electron Microscopy and Energy Dispersive Spettroscopy of the ZnO powder

SEM observations of the ZnO nanopowder (Figure S1) revealed that the particle shape and dimension were not homogeneous; in fact, particles of different shapes were present, with size between 75 nm and 300 nm. Larger agglomerates were also present.

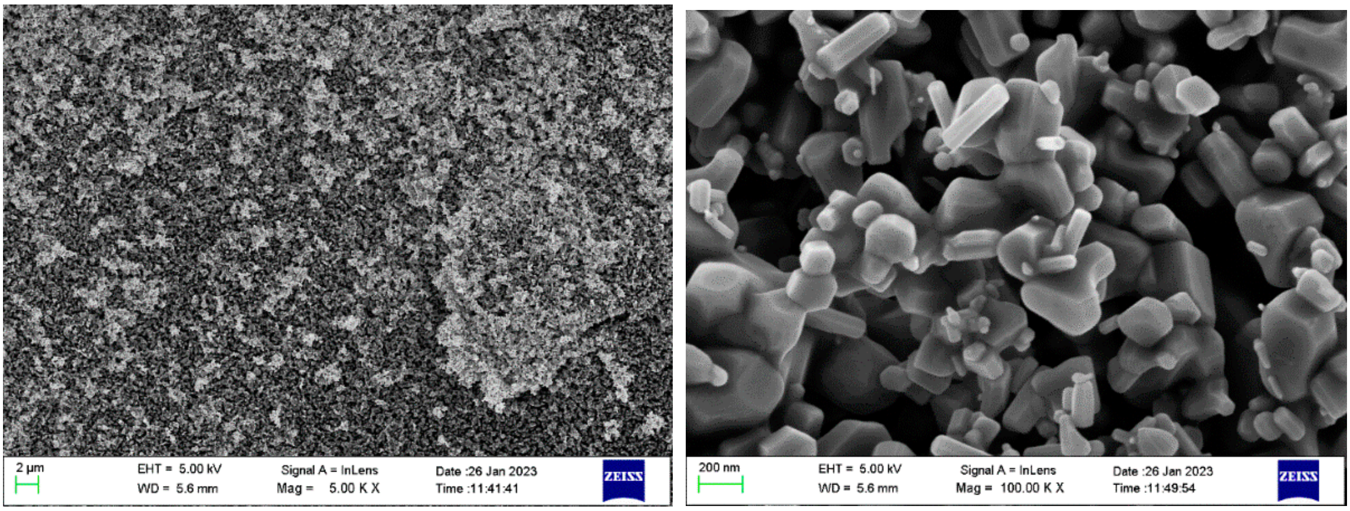

Figure S1. SEM images of ZnO powder: (left) low magnification (5.00 K X) - particle dimension were not homogeneous with also the presence of clusters; (right) high magnification (100.00 K X) - particles of different shapes and size within the nanopowder.

In the EDS microanalysis spectrum obtained from the ZnO nanopowder (Figure S2), the peaks relating to Zinc and Oxygen are highlighted. The spectrum confirms the absence of impurities, thus determining the good quality of the powder. The elements percentage evaluation from the microanalysis is shown in the Table S1. Data confirms the presence of Zinc oxide, with an excess of oxide.

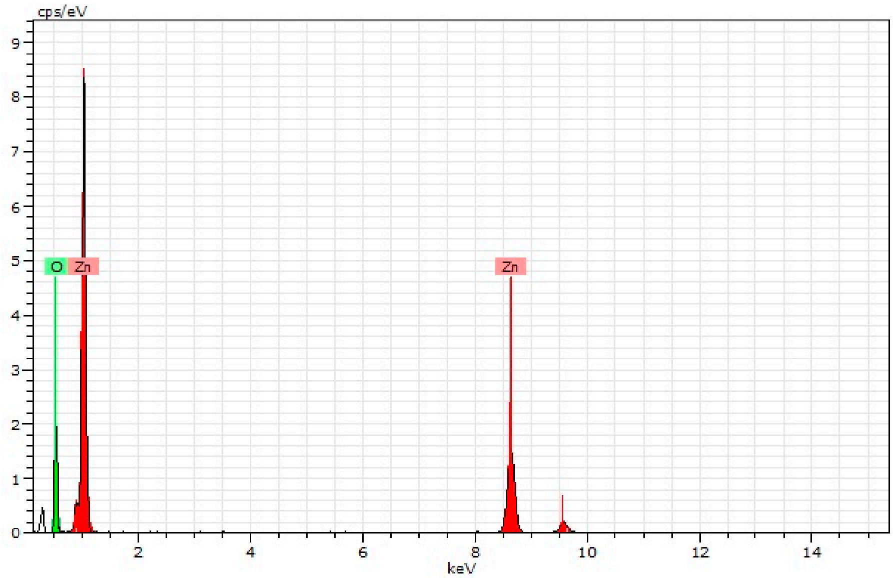

Figure S2. EDS microanalysis of the ZnO powder.

Table S1. EDS microanalysis data of ZnO powder.

Elements	[wt %]	[at %]
**Zinc (Zn)**	74.27	41.39
**Oxygen (O)**	25.73	58.61

X-Ray Diffraction analysis of Zinc Oxide powder

Zinc Oxide powder (ZnO) was also analyzed by a Bruker AXS D8 Advance X-Ray Diffractometer (XRD), in Bragg-Brentano configuration, in order to test the crystallographic quality. The spectrum (Figure S3), in the ϑ-2ϑ range, showed the peaks relating to Zinc Oxide.

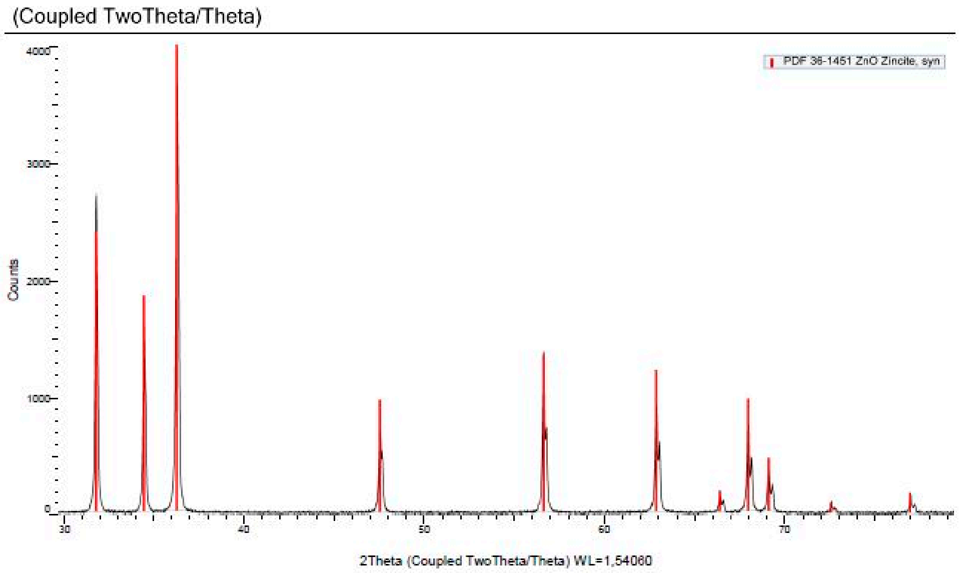

Figure S3. XRD spectrum of the ZnO powder. The sequence and the position of the peaks perfectly match the standard pattern of the Zinc Oxide. The presence of the kα_2 as a double peak in the higher position highlights the good crystallinity of the powder.

XRD pattern put in evidence the crystallinity of the ZnO and the absence of other compounds. In fact, the measured peaks satisfactorily meet the theoretical peaks from the PDF 36-1451. The results put in evidence the good quality of the ZnO powder; the presence of the kα_2 peak close to the main peaks at high degrees highlighted the good crystallization of the sample; indeed, the crystallinity was so high that the crystalline substance acted as X-Ray monochromator splitting the wavelengths present in the beam. This confirms the good quality of the raw powder.
